# THE EMERITUS ORLANDO MARQUES VIEIRA

**DOI:** 10.1590/0100-6991e-2026005825-en

**Published:** 2026-05-22

**Authors:** JOSÉ EDUARDO FERREIRA MANSO

**Affiliations:** 1 - Programa de Pós-Graduação em Ciências Cirúrgicas - Departamento de Cirurgia - Faculdade de Medicina - Universidade Federal do Rio de Janeiro (UFRJ) - Rio de Janeiro - RJ - Brasil

## INTRODUCTION

**Figure 1:**
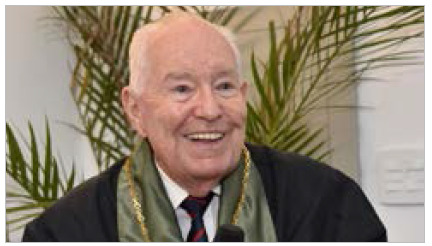
ECBC Orlando Marques Vieira

It was in 1932 when, on November 29, the Brazilian Surgery exponent-to-be Orlando Marques Vieira (Figure 1) was born in Rio de Janeiro, in the Tijuca area, son of Antonio Marques Vieira and Maria Rosa de Almeida Vieira. He married Marinete Neves Marques Vieira and they had two daughters: Fernanda Marques Vieira and Marcela Marques Vieira, both university professors. A curiosity: whenever asked about his birthplace, Prof. Orlando emphatically stated that he was a “Carioca da Gema” (“a true-blood Carioca”), that is, born in the municipality of Rio de Janeiro, son of parents also born in Rio de Janeiro. It should be noted that he was an enthusiastic fan of the soccer team América Futebol Clube.

He began his studies in the kindergarten of the Lafayette Institute and remained in this prestigious educational institution until the conclusion of high school. In his conversations, Prof. Orlando showed immense pride in this group that studied together until the end of pre-college years. The reason: five students from this class became full professors at universities in our country.

He entered the Faculty of Medicine of the University of Brazil (currently the Faculty of Medicine of the Federal University of Rio de Janeiro - UFRJ) in 1951. In 1956, while still an undergraduate student, he was admitted by competitive examination to attend his internship in the Third Chair of the Surgical Clinic, then headed by the eminent professor Mariano Augusto de Andrade. He always said that “since he was a child, he wanted to be a doctor and a professor of medicine” and stated that he did not understand the reason for this thought. Referring to this fact, he uttered the following words during his inaugural speech as Professor Emeritus of UFRJ: “At that moment, I began my university career so cherished since the school benches”. At the end of 1956, at the age of 24, he graduated from the Faculty of Medicine of UFRJ.

After internship, he completed his residency in surgery also in the Third Chair of the Surgical Clinic. After finishing his residency in General Surgery, he was admitted as an instructor of Surgery in the same department. Once the residency was finished, “I needed to find a source of income”. He then applied to the Ministry of Health to practice clinical medicine. He held several positions in the Hospital Organization Division of the Ministry of Health. Later, in 1965, after competitive examinations, he received the titles of Doctor of Medicine and of Associate Professor in General Surgery at UFRJ. In 1969, reinforcing his interest in university life, Prof. Orlando applied for and was approved in the civil service examination for Associate Professor held by the State University of Rio de Janeiro (UERJ) and received the title of Associate Professor in Surgical Clinic.

At FM-UFRJ he participated in several Commissions and examining boards. He was Deputy Director of Graduate Studies from 1992 to 1995 and Head of the Surgery Service of the Clementino Fraga Filho University Hospital, from 1994 to 2002.

In 1989, he applied to participate in the examination for Full Professor of the Department of Surgery at FM-UFRJ. Upon excellent performance, he was approved as a Full Professor of the Department, in the field of Operative Technique. His inauguration was held on June 14, 1989.

His was the name of the 2002 graduating class of Medicine-UFRJ. He was also Patron of the 2004 class and Commencement Speaker of the 2005 class. In 1994, he was the professor chosen to welcome the new residents of the Clementino Fraga Filho University Hospital (HUCFF) - UFRJ. 

**Figure 2:**
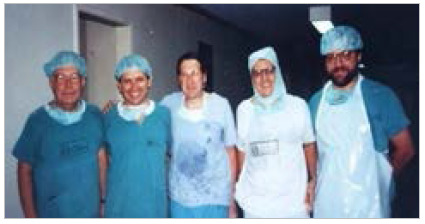
ECBC Oralndo Marques Vieira, TCBC Eduardo Crema, Prof. Laurent Hannoun (Hôpital Pitié Salpêtrière - France), TCBC Vinícius Gomes da Silveira, and TCBC Joaquim Ribeiro Filho.

He stood out in the teaching and practice of General Surgery and, especially, in Gastroenterological Surgery^1^ ^-^ ^3^, including Experimental Surgery^4^, where, during the late 1960s, he stimulated the liver transplant program in dogs, conducted by the then resident and future Professor of the Department of Surgery at UFRJ, Prof. Henrique Murad. Approximately 30 years later, in 1993, the first liver transplant in Rio de Janeiro was performed at the HUFFF. Once again, Prof. Orlando Marques Vieira played a leading role, collaborating and encouraging Prof. Joaquim Ribeiro Filho, who conducted this iconic surgical act (Figure 2). Prof. Orlando also stood out for his work in the surgical treatment of portal hypertension^5^ and diseases of the esophagus and stomach.

Prof. Orlando participated in the authorship of many books, among which we can mention: Surgical Clinic - Theoretical and Practical Foundations^8^: edited for undergraduate and graduate students. Its content addresses in a clear and detailed way the fundamentals of surgical art, including chapters that deal with the scientific basis of surgery and molecular biology; Remember to Live9: a collection of speeches given and selected by Prof. Orlando during his academic life at the Federal University of Rio de Janeiro, at the Brazilian College of Surgeons, and at the National Academy of Medicine; Surgical Clinic: Theory and Practice^10^: written by UFRJ professors, offers a comprehensive introduction to the fundamentals of surgery, with a focus on medical education and student training. It presents a didactic structure, with plain language, updated content, and rich visual material. Each chapter systematically explores diagnosis, treatment, operative techniques, and clinical outcomes, providing a solid foundation for surgical learning; Conducts in Gastric, Biliary, Hepatic, Pancreatic, Endocrine, and Esophageal Surgery1: originating from daily medical practice, this book brings together observations and standardized conducts, consolidated by consensus in surgical evaluation meetings. Its content guides surgical performance, focusing on the applicability and standardization of procedures.

Our professor retired due to statutory imperatives in 2002. In the meantime, he continued, voluntarily, conducting didactic activities in the Department of Surgery of the Faculty of Medicine and assisting in the HUCFF Surgery Service, heading the Hepato-Gastric Surgery section. On June 24, 2005, he received the Title of Professor Emeritus of the Federal University of Rio de Janeiro, granted by the UFRJ University Council. 

He practiced for several years, acting as an on-call physician in the emergency sector of the Paulino Werneck Hospital. In 1985, he was appointed Head of the Surgery Service of that institution. He remained in the position until 1992.

Prof. Orlando Marques Vieira began his distinguished career at the Brazilian College of Surgeons in 1962, as an Associate Member, became a Full Member in 1967, and an Emeritus Member in 2006. He actively participated in several positions in various directories: 28th (1989/1991) First Vice-President; 29th (1992/1994) President; 30th (1995/1997) President of the previous Fiscal Year (1992/1994); 31st (1998/1999) Director of Library and Museum; 32nd (2000/2001) Director of Assets and Headquarters; 33rd (2002/2003) Director of Assets and Headquarters; Member of the Electoral Commission (2018/2019 and 2022/2023).

Later, he became a Natural Counselor of the Superior Advisory Council and an Effective Member of the Permanent Special Commission.

The nationalization of the Brazilian College of Surgeons (CBC), a thought that germinated since the initial boards of our organization, gained new momentum after Prof. Orlando presidency (1992-1994). In addition to his intention to expand the CBC throughout the national territory, he already had extensive experience in the decentralization of the entity. His previous activities in the Continuing Education Commission during the administration of Ruy Ferreira Santos and as vice-president of the Central Nucleus under the leadership of Guilherme Eurico Bastos da Cunha strengthened his commitment to national integration. Under his management, he promoted the creation of new regional chapters and encouraged the reformulation of the Statute of the Brazilian College of Surgeons. These essential reformulations allowed any state to nominate its regional vice-president. 

A significant measure involved altering the frequency of Brazilian congresses from triennial to biennial sessions. Additionally, steps were implemented regarding the mandates of the National Directory. Commenting on these changes, Prof. Orlando stated that they “in addition to modernizing management, provided greater national integration, allowing CBC members from all over Brazil greater participation in the activities of the Brazilian College of Surgeons”.

Professor Orlando Marques Vieira also created the Regional Forum for Research in Surgery and instituted the Alfredo Monteiro, Ruy Ferreira Santos, and Mariano de Andrade Awards, granted to the best works presented at the Forum. He created the Update Course in General Surgery, in 1986, which received surgeons from all over the country for theoretical classes and visits to the main surgical services in Rio de Janeiro. The course was free and the only requirement was that the candidate had to have a minimum of six years of experience as a surgeon.

During his administration, a committee was formed to discuss establishing professional defense, upholding professional dignity, and seeking annuity exemptions for members over 70 with at least 20 years of membership in any category. Currently, associate members over 65 years of age and more than 25 years as an associate are exempt from the annual fee.

Prof. Orlando also implemented a cultural project at the CBC headquarters to hold choir contests, painting exhibitions, and classical music presentations, which were part of the entity’s scientific and social events program.

Emeritus Orlando Marques Vieira, Natural Member of the Superior Advisory Board, in recognition of his dedication, his administrative and educational activities, and the care of his publications, received the following awards: “Brazilian College of Surgeons” (2004), the highest honor offered by the Brazilian College of Surgeons, granted to those who contributed to teaching, progress, and development of Surgery. In 1984, he received the “José de Mendonça” Award, awarded annually to the author whose article addresses issues of surgical technique, published in the Journal of the Brazilian College of Surgeons. In 2004 he was deserving of the “Oscar Alves” Award, awarded annually to the author with the best article published in the Journal of the Brazilian College of Surgeons.

**Figure 3:**
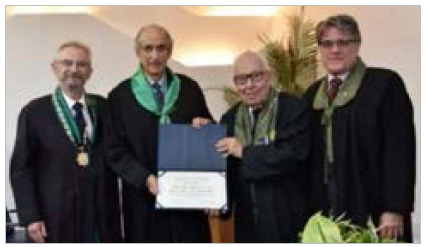
TCBC Luiz Carlos von Bahten, ECBC Samir Rasslan, ECBC Orlando Marques Vieira, TCBC Pedro Eder Portari Filho.

Honoring Emeritus Orlando Marques Vieira, the CBC instituted the award “Magna Conference: Orlando Marques Vieira”, which should be presented, as a mandatory act, during the anniversary ceremonies of the CBC. The first guest to deliver the “Magna Conference: Orlando Marques Vieira” was ECBC Samir Rasslan (Figure 3).

For a long time, Prof. Orlando expressed concern with the training of surgeons and the creation of specialties^10^ ^,^ ^11^, emphasizing the relevance of scientific research and experimental surgery as fundamental components of this training process. In the same way, concerned with the exercise of medical activity and professional dignity, our master already warned, more than thirty years ago, that “the Hippocratic doctor-patient relationship has been replaced by the user-company relationship”. And concluded: “The new commercial parameters of medicine are being governed by companies. sometimes to the detriment of professional activity”^9^.

He took office as a Full Member of the National Academy of Medicine on April 7, 1995. For his candidacy for that position, he presented a memorandum entitled “Surgery of Incomplete Vagotomy”. In this renowned institution, he held the positions of Treasurer (1997-1999), Secretary General (1999-2001; 2003-2005), and Vice-President (2007-2009). He gave 13 speeches at solemn sessions for the inauguration of new academics^9^, for the institution’s anniversaries, and for tributes to academics. 

In an interview with the Virtual Memory Project of the National Academy of Medicine^12^, Prof. Orlando recalled a remarkable episode in his career as a surgeon: in partnership with Prof. Sérgio Andrade, he diagnosed and operated on a patient with Zollinger-Ellison syndrome in 1968. According to Prof. Orlando, this was the first patient diagnosed with this syndrome and submitted to surgical treatment in Brazil.

On November 22, 2023, he was honored with “The International Symposium on Surgery: 90 years of Acad. Orlando Marques Vieira”^13^. The symposium was organized by the Academics Francisco Sampaio, Pietro Novellino, and Rossano Fiorelli.

In a recent meeting, held on September 25, 2025, at the National Academy of Medicine^14^, the opening was dedicated to the Academic and Professor Orlando Marques Vieira, in a “session of nostalgia” that brought together family, friends, and professional colleagues. Professor Orlando was remembered for his contribution to medicine, for his generosity, and for his sense of humor. 

Although prof. Orlando Marques Vieira has developed an intense and fruitful activity at the Faculty of Medicine of UFRJ (Department of Surgery), at the Brazilian College of Surgeons, and at the National Academy of Medicine, mainly in the first two institutions, he also participated in several other houses of culture of medical science: Founding Member of the Academy of Medicine of Rio de Janeiro; Full Member of the Collegium Internationale Chirurgiae Digestivae; Honorary Member of the Academy of Medicine of Amazonas; Corresponding Member of the Royal Academy of Medicine of Spain; Member of the International College of Surgeons; Member of the Board of Trustees of the “State Foundation of General Hospitals of Urgency and Emergency”, established in April 2009; Member of the Technical Chamber of General Surgery and Trauma at the Regional Council of Medicine of Rio de Janeiro (2008-2009); and President of the Brazilian Society of the History of Medicine - Chapter of the State of Rio de Janeiro (SBHM-RJ).

Prof. Orlando Marques Vieira left a remarkable legacy in the training of generations of students, many of whom became outstanding surgeons and professors. Convinced of the relevance of continuous improvement, he vigorously defended the integration between scientific research and surgical training, pillars that he considered indispensable for medical excellence. He was also a firm defender of the valorization and protection of the profession, always attentive to the causes that strengthened the dignity of the physician, as well as a great supporter of the expansion of the Brazilian College of Surgeons throughout the national territory.

Prof. Orlando Marques Vieira lived intensely with his family, and in parallel with his professional life, until December 28, 2023, when he left to live eternally.

I would like to thank TCBC Ramiro Colleoni Neto, Director of Publications at the Brazilian College of Surgeons, for the gracious invitation to write about the distinguished Professor Orlando Marques Vieira, whom I was privileged to meet during my surgical internship in 1974.
